# Brazilian Red Propolis: Extracts Production, Physicochemical Characterization, and Cytotoxicity Profile for Antitumor Activity

**DOI:** 10.3390/biom10050726

**Published:** 2020-05-06

**Authors:** Felipe Mendes de Andrade de Carvalho, Jaderson Kleveston Schneider, Carla Viviane Freitas de Jesus, Luciana Nalone de Andrade, Ricardo Guimarães Amaral, Jorge Maurício David, Laíza Canielas Krause, Patrícia Severino, Cleide Mara Faria Soares, Elina Caramão Bastos, Francine Ferreira Padilha, Silvana Vieira Flores Gomes, Raffaele Capasso, Antonello Santini, Eliana Barbosa Souto, Ricardo Luiz Cavalcanti de Albuquerque-Júnior

**Affiliations:** 1 Tiradentes University (UNIT), Av. Murilo Dantas, 300, Aracaju 49010-390, Brazil; felipemdadc@gmail.com (F.M.d.A.d.C.); jadersonqmc@gmail.com (J.K.S.); carlavfj@gmail.com (C.V.F.d.J.); laiza_canielas@hotmail.com (L.C.K.); pattypharma@gmail.com (P.S.); cleide18@yahoo.com.br (C.M.F.S.); elina@ufrgs.br (E.C.B.); fpadilha@yahoo.com (F.F.P.); svfloresta@hotmail.com (S.V.F.G.); 2 Institute of Technology and Research (ITP), Av. Murilo Dantas, 300, Aracaju 49032-490, Brazil; 3 Federal University of Sergipe (UFS), Avenida Marechal Rondon, São Cristovão 49100-000, Brazil; luciana.nalone@hotmail.com (L.N.d.A.); ricardoamaral23@hotmail.com (R.G.A.); 4 Federal University of Bahia (UFBA), Salvador/BA 40170-110, Brazil; jmdavid@ufba.br; 5 Tiradentes Institute, 150 Mt Vernon St, Dorchester, MA 02125, USA; 6 Department of Agricultural Sciences, University of Napoli Federico II, Via Università 100, 80055 Portici, Italy; rafcapas@unina.it; 7 Department of Pharmacy, University of Napoli Federico II, Via D. Montesano 49, 80131 Napoli, Italy; asantini@unina.it; 8 Department of Pharmaceutical Technology, Faculty of Pharmacy, University of Coimbra, Pólo das Ciências da Saúde, Azinhaga de Santa Comba, 3000-548 Coimbra, Portugal; 9 CEB-Centre of Biological Engineering, University of Minho, Campus de Gualtar, 4710-057 Braga, Portugal

**Keywords:** red propolis, extraction, supercritical liquids, antitumor activity

## Abstract

Brazilian red propolis has been proposed as a new source of compounds with cytotoxic activity. Red propolis is a resinous material of vegetal origin, synthesized from the bees of the *Appis mellifera* family, with recognized biological properties. To obtain actives of low polarity and high cytotoxic profile from red propolis, in this work, we proposed a new solvent accelerated extraction method. A complete 2^3^ factorial design was carried out to evaluate the influence of the independent variables or factors (e.g., temperature, number of cycles, and extraction time) on the dependent variable or response (i.e., yield of production). The extracts were analyzed by gas chromatography coupled with mass spectrometry for the identification of chemical compounds. Gas chromatography analysis revealed the presence of hydrocarbons, alcohols, ketones, ethers, and terpenes, such as lupeol, lupenone, and lupeol acetate, in most of the obtained extracts. To evaluate the cytotoxicity profile of the obtained bioactives, the 3-(4,5-dimethyl-2-thiazole)-2,5-diphenyl-2-*H*-tetrazolium bromide colorimetric assay was performed in different tumor cell lines (HCT116 and PC3). The results show that the extract obtained from 70 °C and one cycle of extraction of 10 min exhibited the highest cytotoxic activity against the tested cell lines. The highest yield, however, did not indicate the highest cytotoxic activity, but the optimal extraction conditions were indeed dependent on the temperature (i.e., 70 °C).

## 1. Introduction

Cancer is a group of diseases characterized by disordered growth of cells that invade tissues and organs, with high morbidity and mortality, and represents one of the most fatal diseases worldwide [1]. About 21 million new cancers and 8.2 million cancer deaths worldwide are estimated until 2030 [2]. Surgery, radiotherapy, and chemotherapy are the most used approaches in the management and treatment of the disease [3]. Chemotherapy is commonly associated with the non-specific delivery of antineoplastic drugs capable of inhibiting mitosis, causing cell death and systemic toxicity. The drug targets are not only cancer cells but also healthy cells, thereby causing serious adverse side effects, e.g., diarrhea, hair loss, mucositis, immunosuppression, nausea, and vomiting, thus compromising the patients’ quality of life [4].

In developing and emerging countries, a large part of the population makes use of natural products in the treatment of health conditions, as these products are commonly related to a lower risk of inducing adverse side effects [5]. Propolis is a complex resinous mixture of vegetable origin, synthesized by bees from the collection of various natural substances. Depending on the flora at the collection site, propolis samples may vary in color and chemical composition [6]. A red variety of Brazilian propolis has been described in the last years, with the ethanolic extracts being enriched in isoflavones, mainly formononetin, daidzein, and biochanin A [7]. Studies have demonstrated that such hydroalcoholic and ethanolic extracts have potential antitumoral activity both in vitro [8,9] and in vivo [10,11,12,13,14,15].

In addition to the phenolic compounds often extracted with high-polar solvents, low-polar chemical constituents have also been described in samples of red propolis, including terpenes and others [6]. Some of these chemical compounds, such as methyl eugenol, a methylated ether previously reported as one the major constituents of the essential oil of red propolis, have been demonstrated to induce anticancer activity [16]. Furthermore, other substances of lower polarity present in samples of red propolis, such as sesquiterpenes, have also exhibited antitumor activity in vitro [17,18].

The solvent accelerated extraction (SAE) method is a fast process that uses solvent under high pressure to promote greater desorption of the analytes present in the matrix, offering less exposure to organic solvents while producing more selective extracts [19]. Pressure liquid extraction (PLE) has been successfully used to obtain compounds of low polarity from propolis samples [19,20]. Although high pressurized ethanol (in supercritical state) has been previously applied to extract chemical compounds from Brazilian red propolis [21], the use of PLE with low-polarity solvents (such as hexane) to extract chemical compounds with potential anticancer activity has not been studied so far. The aim of this study was to chemically characterize hexane extracts of Brazilian red propolis obtained using SAE and assess their cytotoxic activity against HCT116 and PC-3 cell lines derived from human colorectal carcinoma and prostate small cell carcinoma, respectively.

## 2. Materials and Methods

### 2.1. Collection and Processing of Red Propolis

The red propolis was obtained from the Santo Antônio apiary located in the municipality of Barra de Santo Antônio, Alagoas, Brazil, under coordinates 09°24′17” S 35°30′26”, in August 2017. The collected propolis samples were crushed in a grinder (Cadence, Piçarras, SC, Brazil) and then sieved (60 mesh) to obtain an adequate granulometry (approximately 0.250 mm), in order to increase the surface area and improve the homogenization of the starting material in the extraction process. The sample was then stored in a polyethylene container sealed and refrigerated between 2 and 8 °C.

### 2.2. Extraction by Pressurized Liquids

The extraction process was performed using a Dionex ASE™ 100 extractor (Dionex, Sunnyvale, CA, USA), with a 34-mL capacity stainless steel extraction chamber, a rinse bottle, and glass flasks with a capacity of 250 mL.

### 2.3. Full 2^3^ Factorial Design Experiments

A complete 2^3^ factorial design approach (Table 1) of three independent variables or factors (i.e., temperature, number of cycles, and extraction time) and three experimental levels (center point) was used to define the stage of preparation of the extract, totaling 11 trials. In this study, the levels were coded at the minimum (−1), central (0), and maximum (+1). The planning of Table 1 was performed, and the assay response was evaluated in terms of the extract yield (%). 

For each extract, 10 g of the crude propolis were placed into the extraction chamber, hermetically sealed, and inserted into the equipment. The independent variables were adjusted in the extraction equipment, namely, a constant pressure of 1450 psi (100 bar) exerted by pneumatic pressurization by nitrogen (N_2_), flushing of 100%, purge of 60 s, and hexane as the solvent. Each extract was concentrated under reduced pressure on a rotary evaporator (RE801BW2, Yamato Scientific America Inc, Santa Clara, CA, USA) for 10 min at 55 °C. The obtained extract was then taken to a laminar flow oven for 72 h at a constant temperature of 45 °C. The dry extracts were passed through a thermogravimetry scale to determine the humidity and then stored at room temperature for further analysis. Experimental planning and analysis of the results were performed using Statistica Software 7.0 (Statsoft^®^, Tulsa, USA). The significance of the effects related to the studied variables was evaluated by analysis of variance (ANOVA) for each response and the effects were verified using *p* values less than 0.05 to indicate that the variable was significant within the experimental domain studied, with a confidence level of 95%.

### 2.4. Yield of Production

The yield of the extracted material was determined based on the percentage of mass obtained, using the initial mass of the propolis before the extraction and the material obtained after extraction and removal of the solvent as reference, according to the following equation:

$$Yield\;\left(\%\right)=\frac{EM}{IM}\times 100,$$

where *EM* stands for the extract mass (g) and *IM* for the initial mass (g).

### 2.5. Gas Chromatography Coupled with Mass Spectrometry (GC/MS)

The extracts of red propolis were dissolved in 5 mL of distilled hexane. Samples were filtered on 0.45-μm nylon membranes (Merck KGaA, Darmstadt, Germany) prior to being injected into CG/qMS (QP2010 Plus, Shimadzu, Kyoto, Japan). Analyses were conducted on an Agilent capillary column coated with DB-5 (5% diphenyl and 95% dimethylpolysiloxane, 30 m × 0.25 mm ID, 0.25 µm film thickness) (Palo Alto, CA, USA). The flow rate of the carrier gas (He) was 1 mL/min. The injection mode was splitless in the automatic injector AOC20i, and the temperature of the injector, interface, and detector were 300 °C. The column temperature ramp was maintained at 40 °C for 1 min, and raised 3 °C/min to 300 °C (kept for 15 min). The analysis mode was scan mode (45 to 500 Da). All data were treated by the GCMS Solution software 2.6.1 (Shimadzu, Kyoto, Japan), using a signal/noise of 3. The compounds were tentatively identified using the spectral library database of the National Institute of Standards and Technology (NIST, Beta release STRBase 2.0). For the identification, the spectra comparison and detailed analysis of the MS spectra and position (retention time) in the chromatogram were used. Only the peaks where the similarity with the libraries was higher than 80% were identified, together with confirmation through the linear temperature programmed retention index (LTPRI) (considering a difference of up to 20 units).

A C6-C30 mixture of n-alkanes (1 μL) at the concentration of 10 mg/L was injected into the GC/qMS system under the same conditions used for the samples to calculate the LTPRI. We used the retention time of each standard n-alkane to carry out this analysis, since this is a quantitative parameter. The retention indices were calculated for each compound in the sample and the values were compared to those from the literature for the same column (DB-5 or similar).

### 2.6. In Vitro Cytotoxicity Analysis

To determine the cytotoxic effect of the obtained extracts against tumor cell lines, the method of the 3-(4,5-dimethyl-2-thiazole)-2,5-diphenyl-2-H-tetrazolium bromide (MTT) (Sigma Aldrich, Brazil) salt was used [22]. For all experiments, in 96-well plates, tumor cell lines were plated (100 μL/well) at concentrations of 0.7 × 10^5^ cells/mL HCT116 (colon cancer) and 1 × 10^5^ cells/mL of PC3 cells/mL (prostate cancer). All cells were obtained from Fundação Oswaldo Cruz (Fiocruz, Brazil). The cell lines were selected based on the worldwide incidence of these two types of cancer. After 24 h, the extracts were dissolved in 0.3% dimethyl sulfoxide (DMSO) and added to each well in a single concentration of 50 μg/mL. The experiment was performed three independent times in triplicate, using doxorubicin 0.25 μg/mL and 0.3% DMSO as the positive and negative control, respectively. The plates were incubated for 72 h in a 5% CO_2_ oven at 37 °C. After incubation, the plates were centrifuged (15 g/15 min) at 4 °C and the supernatants were removed. Subsequently, 150 μL of the MTT solution (0.5 mg/mL) were added to the wells and the plates were incubated for 3 h. Following this period, the plates were again centrifuged (30 g/10 min) at 4 °C, the supernatants were discarded, and the precipitate was resuspended in 150 μL of pure sterile DMSO. For the quantification of formazan crystals formed by viable cells, the absorbance was read using a multi-plate reader (DTX 880 Multimode Detector, Beckman Coulter Inc., Packard, ON, Canada) at 595 nm. All values were converted to the percent inhibition of cell growth (% *ICG*) by the following equation:

(1) $$ICG\%=100-\left[\frac{T}{CN}\right]\times 100,$$

where *T* is the absorbance of the red propolis extract and *CN* is the absorbance of the negative control.

For each tested cell line, the results were analyzed using an *ICG*% scale as follows: (i) Samples of low activity have *ICG* ≤ 50%, (ii) samples with moderate activity have 50% < *ICG* < 75%, and (iii) samples of high activity have *ICG* ≥ 75%. To determine the minimum inhibitory concentration (IC50, capable of causing 50% of its maximal effect), the same protocol as described for the determination of *ICG*%, was performed in the same cell lines but by varying the extract concentration (0.39–50 μg/mL) only. The percentage of cell death was analyzed according to the mean ± standard error. All the assays were carried out in triplicate. The percentage of inhibition x log of the concentration was recorded and their IC50 and their respective confidence intervals (IC 95%) were recorded from non-linear regression. The analyzes and graphs were elaborated using the GraphPad Prism version 7.0 software (San Diego, CA, USA).

### 2.7. Statistical Analysis

The quantitative variables, expressed as the mean ± standard error of the mean, were subjected to the test of homoscedasticity and normal distribution of values. For the normality test, the Shapiro–Wilk test was applied, whereas the Bartlett (assuming that the variable has a normal distribution) and Levene (assuming that the data have a continuous distribution) tests were applied for homoscedasticity analysis. Data with a normal distribution and homoscedasticity were compared using the ANOVA test, followed by post hoc Tukey’s multiple comparisons test. Data that did not obey these assumptions (normal distribution and/or homoscedasticity) were compared using the Kruskal–Wallis test with post hoc Dunn’s multiple comparisons test. Differences between means were considered significant when *p* values were lower than 0.05.

## 3. Results and Discussion

The extraction processes of chemical compounds from propolis samples commonly use ethanol, water, hexane, ethyl acetate, or chloroform as solvents. However, these conventional extraction processes have some drawbacks, such as a strong residual taste, risk of harmful effects to the environment, poor quality of the obtained extract, and the need for a long extraction time. High pressure has been applied in the extraction, fractionation, refinement, and deodorization of natural sample matrices at the laboratory scale and industrial scale, with supercritical CO_2_ and ethanol (usually used as a co-solvent) being the most frequently used solvents [23]. 

**Figure 1:**
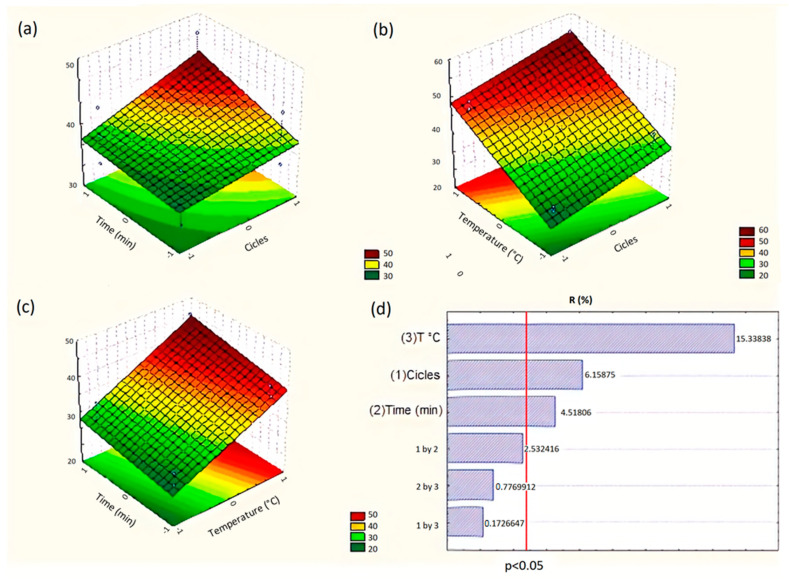
(**a**) Surface response chart of the 2^3^ factorial design, representing the interactions of two distinct factors (time and cycle) favoring the determination of the best extraction conditions. (**b**) Surface response chart representing the interactions between the temperature and number of cycles. (**c**) Surface response chart representing the interactions between the time and temperature variables. (**d**) Pareto graph of 2 interactions between the variables applied to determine the yield as a response of the developed factorial planning. The vertical interrupted red line (in d) represents the significance of the compared variables; the right-hand side of the cut has significant representativity and the left-hand side does not present significance.

In this work, a full 2^3^ factorial design was applied for the optimization of the extraction method of substances of low polarity from red propolis, aiming to be fast, of low expenditures, and high yield. This model allows a combination of three variables and one central point with three samples and is frequently used in optimization studies [24,25,26,27,28]. However, studies applying factorial design for the analysis of extractive processes of propolis samples using pressurized liquids have not yet been reported in the literature. The results of the comparisons between the variables tested to obtain the best yields are shown in Figure 1. The surface responses show that the temperature, cycles, and time significantly influenced the yields (*p* < 0.05) but not the combination of variables (*p* > 0.05).

It has been reported that different extraction conditions can generate different responses with respect to their yield and their chemical composition. Thus, yield can be considered an important response variable to be evaluated in the research of different methods of natural product extraction [29]. For the extraction of low-polarity compounds, low-polarity solvents are also used because of the higher affinity promoted by the interaction between the polarities of the solute and the solvent, which favors the dragging of similar substances. Among the solvents with these chemical characteristics, hexane has been selected. This solvent has been frequently used for extractions of compounds of low polarity [30]. In the current study, there was large variability in the yield of the extractive processes within the different conditions tested, varying from 18.2% to 64.63%. The purpose of the experimental planning used in this study was to develop the extractive process based on yield, with optimal conditions assumed as those that provided values equal to or greater than 11%. Due to the lack of similar studies in the literature reporting extracts of low polarity with red propolis, this percentage (11%) was based on the reference value suggested by the Brazilian Ministry of Agriculture for hydroethanolic and ethanolic extracts of green propolis samples [31].

**Figure 2:**
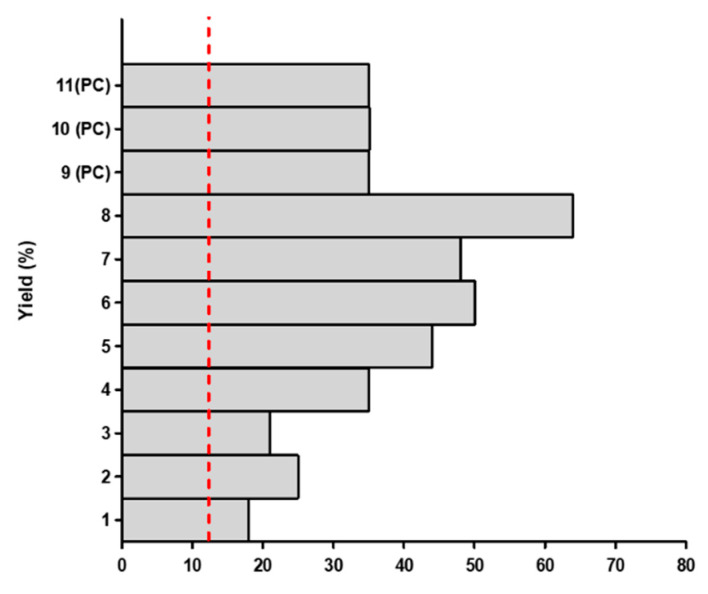
Evaluation of the yield of red propolis extract samples obtained by the solvent accelerated extraction method, where the vertical interrupted red line represents the cutoff of 11%.

As shown in Figure 2, the yield obtained in all the samples ranged from 18.34% to 64.63%. The yields obtained in the current study were considered satisfactory in comparison to those reported by Biscaia and Ferreira [20] (10.79%), Zordi et al. [32] (24.8%), and Machado et al. [33] (9.16%), using supercritical fluid extraction, with CO_2_ and ethanol as the co-solvent. However, none of the extraction procedures described above were performed within the conditions tested in the current study, which makes it difficult to establish a proper comparison and deeper analysis of these data.

In the present study, the highest yields were obtained at 70 °C. These results can be explained by the resinous nature of propolis. When this heterogeneous substance is subjected to high temperature and pressure, the material of low polarity can be solubilized and dragged together with the compounds of greater affinity with the low-polarity solvent [34]. Herrero et al. used an experimental design for antioxidant extraction from *Spirulina platensis* microalgae by ASE, using hexane, petroleum ether, ethanol, and water [35]. The authors reported that although a similar antioxidant capacity could be seen for the extracts obtained using the organic solvents, a slightly higher antioxidant profile was recorded with hexane-based extracts. This was attributed to the capacity of hexane to extract a higher amount of non-polar compounds (carotenoids, among others) that can be extracted using hexane, which increase the antioxidant activity. 

Gas chromatography is often used in the characterization of the chemical compounds of natural products. This technique is usually employed to separate compounds that have low polarity and high volatility. These characteristics, associated with the availability of a large library of mass spectra information of each substance, allow the identification of the chemical compounds present in the samples [16,36]. Due to the advantages of this separation technique, and the low polarity of the extracts, gas chromatography coupled with mass spectrometry (GC/MS) was used in this study. Chromatograms with similar profiles were obtained for all extracts, regardless of the tested conditions. A total of 46 compounds were identified and are shown in Table 2. The identification was confirmed from the comparison with the retention index of the peaks, using the NIST Chemistry WebBook, SRD 69 as a reference.

A wide range of chemical compounds with no previously reported biological activity were found in the extracts of red propolis. The presence of aromatic hydrocarbons may be related to the contamination of the water and soil of the apicultural grass (collection sites of the bees), mainly by fuels, since these compounds are often found around fuel stations and account for a considerable part of air pollution [37]. On the other hand, unsaturated hydrocarbons, such as heptacos-1-ene and nonacos-1-ene, belonging to the family of alcohols, are often found in products derived from the fermentation of sugar in plants [38]. Regarding the chemical compounds belonging to the class of ethers, only methyl eugenol has been previously reported to show biological activity, as a promising agent against hemorrhoids, rheumatism, contusions, and seizures. Two compounds belonging to the class of ketones, 2-(3H)-furanone, 5-dodecyldihydro-, which do not present biological activity, and acetophenone, which presents anti-inflammatory and antioxidant activities [5]. Additionally, according to Favier et al. [39], acetophenone is a phenolic compound that represents a secondary metabolite often found in plants subjected to environmental stress.

Among the chemical compounds found in RPE presenting well-established biological activity, there were the terpenes lupeol (C_30_H_50_O), lupeol acetate (C_32_H_52_O_2_), and lupenone (C_30_H_48_O). Triterpenoids have been demonstrated to exert cytotoxic effects on human hepatocellular carcinoma (Hep-G2) and melanoma (MEL-2) cell lines by the inhibition of topoisomerase II and farnesyl transferase protein [40]. Moreover, triterpenes have been reported to reduce the number of aberrant cells, micronucleus DNA, and morphological signs of cytotoxicity (e.g., pyknosis, caryolysis, and karyorrhexis) in a benzo [α] pyrene-induced clastogenicity model [41]. Regarding in vivo experimental models, a lupeol-associated antitumor effect on epidermal tumors induced by TPA (12-tetradecanoylphorbol-13-acetate) has been previously reported, likely due to modulation of the NF-κB and PI3K/Akt pathways [42]. Furthermore, lupeol applied on the dorsum of rats displayed chemopreventive effects on the carcinogenesis induced by 7,12-dimethylbenz (a) anthracene (DMBA) [43]. These data suggest that substances presenting those compounds in their chemical composition, such as the RPE obtained in the current study, might display cytotoxicity or even antitumor activity.

**Figure 3:**
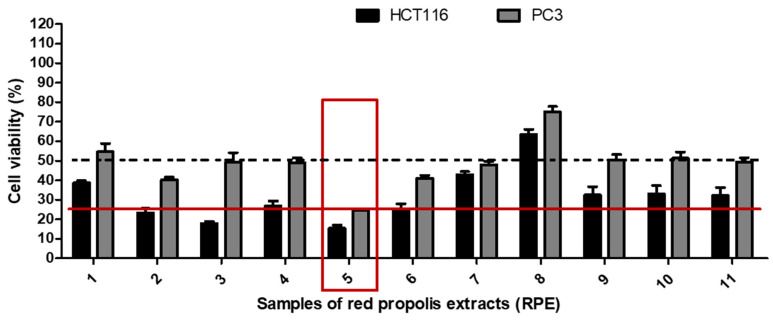
Cytotoxic analysis against tumor cell lines derived from human malignant neoplasms HCT116 (colon adenocarcinoma) and PC3 (prostate adenocarcinoma). - - - - Minimum percentage limit of cytotoxic activity considered to be at least moderate. **^______^** Minimal percentage limit of cytotoxic activity considered strong.

For screening purposes, the cytotoxic activity of a given product against tumor cell lines, either a plant extract or an isolated molecule, is considered moderate when the viability index is between 25% and 50% and strong when this index is less than 25%, whereas cell viability above 50% means no consistent cytotoxic activity. In the current study, the cytotoxic potential of the 11 samples was assessed using two different tumor cell lines, colon cancer (HCT116) and prostate (PC3). The choice for these cell lines was based on the high frequency of colon and prostate cancers worldwide [44]. As demonstrated in Figure 3, most samples showed at least moderate cytotoxic activity against HCT116 cells, except for sample 8, and against PC3, excluding samples 1 and 8. However, only sample 5 showed strong cytotoxic activity against both tumor cell lines.

The cytotoxic activities of the RPE might be related to the presence of triterpenes in their chemical composition [45]. Supporting this hypothesis, different extracts rich in lupeol and derivatives have been demonstrated to exert cytotoxic activity against several malignant tumor cell lines, including NCI-H292 (lung mucoepidermoid carcinoma), MCF-7 (breast adenocarcinoma), and HEp-2 (squamous cell carcinoma of larynx) [46]. Besides, the use of lupeol has been shown to have potent cytotoxic activity against MCF-7 (breast adenocarcinoma) cells [47], U87MG (glioblastoma), CEM/ADR5000 (leukemia), and HCT116 (colon adenocarcinoma) [48]. On the other hand, lupeol acetate has cytotoxic activity against lung carcinoma (A549), colon adenocarcinoma (DLD-1), breast (MCF-7, MDA-MB468), pancreas (MIAPaCa-2), prostate (C-1) and ovary (SK-OV-3), cervical carcinoma (HeLa), hepatocellular carcinoma (PLC/PRF/5), and renal carcinoma (786-0, Caki-1) [49]. In addition, extracts containing lupenone have presented cytotoxic activity against MCF-7, HT-29 (colon) and HeLa cells (cervix) [50]. Thus, the presence of triterpenes, such as lupeol, lupenone, and lupeol acetate, in the RPE analyzed in the current study, although in apparently low concentrations, the results see to support the moderate cytotoxic activity of the majority of the samples. Furthermore, sample 5 demonstrated stronger cytotoxicity against HCT116 and PC3 cells, which could be explained by the presence of both lupeol and lupeol acetate in the chemical composition of the extract.

**Figure 4:**
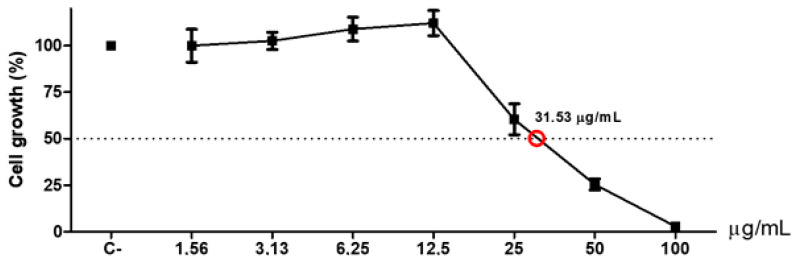
Average inhibitory concentration graph capable of causing 50% of the maximal effect in HCT116 cells.

As sample 5 presented the strongest cytotoxic effects and HCT116 cells were the most sensitive to RPE, they were both selected to the IC 50 assessment assay (mean inhibitory concentration capable of causing 50% of maximal effect), as demonstrated in Figure 4. According to the guidelines for testing for cytotoxicity with plant-derived extracts (Gad-Shayne, 2009), IC 50 < 10 μg/mL is considered to be “very toxic”; IC 50 between 10 and 100 μg/mL would be “potentially toxic”; IC 50 100–1000 μg/mL “potentially harmful”; and IC50 > 1000 μg/mL “potentially non-toxic”. As the IC 50 value obtained in the current study was 31.53 μg/mL, sample 5 could be considered “potentially toxic”, which makes it a target for alternative treatments against cancer, either as chemotherapeutic or chemopreventive agents.

It is possible to estimate some hypotheses about the pathophysiological mechanisms that might be involved in the cytotoxic activity. Previous studies have asserted that the anticancer potential of triterpenes could be dependent on the generation of reactive oxygen species (ROS), which would later lead to mitochondrial membrane rupture and potential apoptosis of tumor cells [49]. Furthermore, triterpenes, such as lupeol, are able to downregulate Bcl-2 and Bcl-xL protein expression, and contribute to the induction of MCF-7 cell death by activating mitochondrial pathway apoptosis [47]. In addition to the activation of the intrinsic pathway of apoptosis by regulating the Bcl-2 family genes’ expression, the involvement of upregulation of proapoptotic proteins, such as Smac/DIABLO, can also be postulated. Smac/DIABLO is a protein that promotes the displacement of the inhibitor of apoptosis protein (XIAP) from caspase 9, thus neutralizing the ability of XIAP to suppress the activity of the effector caspase within the apoptosome complex [51]. In fact, a cytotoxic effect of methanol extract of Ficus Deltoidea (FD2c), rich in phytochemical lupeol, against PC-3 and LNCaP prostate cancer cells, with an IC50 of 29 and 23 ug/mL, respectively, mediated by downregulation of Bcl-2 and upregulation of Smac/DIABLO, and consequent activation of caspases 3, and 7, involved in the intrinsic pathway of apoptosis cascade, has been previously reported [52]. As these IC values are remarkably similar to the IC50 of RPE sample 5 against HCT116 cells (31.53 ug/mL), we speculate whether these two protein families might be involved in the pathogenesis of cytotoxic activity exerted by RPE. However, further studies are still necessary to clarify the biochemical and molecular mechanisms underlying the cytotoxic effects of RPE on tumor cell lines. Besides, in order to be site specific for tumor cells, the loading of such chemotherapeutic and/or chemopreventive agents or their extracts into nanoparticles has been proposed to minimize the risk of cytotoxic events in healthy cells [53,54]. Additional screening tests, such as genotoxicity [55,56], oxidative stress [57], and apoptosis [58], have been proposed.

## 4. Conclusions

In summary, the hexane-based extracts obtained in this study showed very a similar chemical composition, represented by saturated and unsaturated aromatic hydrocarbons, ketones, alcohols, ethers, and terpenes. Most of the extracts exhibited moderate cytotoxic activity. In addition, the extract obtained at 70 °C and using one cycle of 10 min presented the strongest cytotoxic activity against HCT116 and PC3 tumor cell lines, and such activity could be related to the presence of terpenes, such as lupeol and lupeol acetate, in its chemical composition.

## Figures and Tables

**Table 1:** Experimental variables used in the 2^3^ full factorial design.

Factors	Levels		
	Minimum Value (−)	Central Point (0)	Maximum Value (+)
Number of cycles	1	2	3
Extraction time (min)	10	15	20
Temperature (°C)	40	55	70
**Assay**	**Number of cycles**	**Extraction time (min)**	**Temperature (°C)**
1	−1 (1)	−1 (10)	−1 (40)
2	1 (3)	−1 (10)	−1 (40)
3	−1 (1)	1 (20)	−1 (40)
4	1 (3)	1 (20)	−1 (40)
5	−1 (1)	−1 (10)	1 (70)
6	1 (3)	−1 (10)	1 (70)
7	−1 (1)	1 (20)	1 (70)
8	1 (3)	1 (20)	1 (70)
9 (PC)	0 (2)	0 (15)	0 (55)
10 (PC)	0 (2)	0 (15)	0 (55)
11 (PC)	0 (2)	0 (15)	0 (55)

**Table 2:** Compounds identified in the extract of red propolis by gas chromatography coupled with mass spectrometry (GC/MS) with the relative area (%) and linear temperature programmed retention index (LTPRI) of the compounds.

Compounds	Samples											LTPRI		
	1	2	3	4	5	6	7	8	9	10	11			
**Aromatic Hydrocarbons**	**Area (%)**											**Theoretical**	**Calculated**	**Difference**
Toluene	8.0	2.5	2.6	2.1	13.2	3.3	7.8	17.9	-	5.5	4.0	774	774	0
Benzene, 1,2,3-trimethyl-	0.5	6.1	0.6	4.4	-	3.6	2.9	7.2	-	-	2.0	990	992	2
Benzene, 1,2,4-trimethyl-	3.3	2.0	5.4	1.1	8.7	0.9	0.8	1.7	5.3	2.7	0.7	976	976	0
Indane	0.6	0.3	-	-	0.4	-	-	-	-	-	-	1031	1032.4	1,4
Benzene, 1,2-diethyl-	0.3	-	-	-	-	-	-	-	-	-	-	1046	1045	−1
Benzene, 1-methyl-3-propyl-	0.4	0.3	-	-	0.4	-	-	-	-	-	-	1048	1049	1
Benzene, 1,4-diethyl-	0.4	0.5	-	-	0.5	-	-	-	-	-	-	1052	1053	1
Benzene, 1,3-diethyl-	-	-	-	-	0.5	-	-	-	-	-	-	1054	1054	0
Benzene, 2-ethyl-1,4-dimethyl-	0.6	0.2	-	-	0.4	-	-	-	-	-	-	1082	1085.2	4,3
Benzene, 4-ethyl-1,2-dimethyl-	-	-	-	-	0.3	-	-	-	-	-	-	1083	1083	0
Benzene, 1,2,3-trimetoxy-5-(2-propenyl)-	-	-	0.7	0.5	0.4	0.4	0.3	-	-	-	-	1554	1554	0
**Saturated hydrocarbons**	**Area (%)**													
Tricosane	-	-	4.0	2.4	2.0	3.0	0.2	3.0	4.0	3.5	0.4	2299	2300	1
Pentacosane	15.1	10.5	14.5	8.4	6.1	7.9	0.6	10.5	11.1	10.3	0.8	2499	2500	1
Hexacosane	-	0.4	1.4	0.9	1.0	0.8	0.8	0.2	1.5	0.6	0.4	2599	2600	1
Heptacosane	0.2	24.6	25.0	19.1	19.9	-	-	-	-	-	1.2	2701	2700	−1
Octacosane	0.5	1.0	0.7	0.8	0.8	0.7	0.4	1.3	0.4	0.3	0.1	2799	2800	1
Nonacosane	0.1	2.5	1.8	11.9	10.2	16.6	13.0	16.2	21.0	15.8	14.2	2899	2900	1
Triacontane	-	1.5	20.4	0.9	-	0.9	0.7	0.7	1.1	1.0	1.0	2998	3000	2
Untriacontane	0.2	23.0	-	17.6	-	18.5	20.0	22.4	31.0	21.3	19.9	3108	3100	−8
**Unsaturated Hydrocarbons**	**Area (%)**													
Heptacos-1-ene	-	-	-	-	0.3	0.9	2.1	0.3	0.8	0.5	20.2	2674	2684.2	10.2
Nonacos-1-ene	-	-	1.4	1.1	-	-	-	-	-	-	-	2875	2877	2
**Alcohols**	**Area (%)**													
1-Pentanol, 2,3-dimethyl-	-	-	-	-	0.4	-	-	0.7	-	-	-	809	827	18
2-Hexyn-1-ol	-	-	-	-	2.4	-	-	-	-	-	-	846	847	1
1-Butanol, 3-methyl-, acetate	3.2	1.0	1.6	0.9	5.0	1.0	2.4	4.2	2.7	1.5	-	872	872	0
Behenic alcohol	2.1	0.6	-	0.3	-	0.4	7.2	0.4	0.5	0.3	8.6	2473	2470	−3
Octacosanol	-	-	-	3.7	-	4.8	3.8	2.7	1.1	3.7	5.1	3098	3110.6	12.6
1-Heptacosanol	-	-	-	16.0	-	-	15.6	2.1	-	15.8	-	3297	3307	−10
1-Triacontanol	-	-	-	-	-	17.6	-	-	-	-	-	3306	3306	0
**Ethers**	**Area (%)**													
Methyleugenol	-	-	0.6	0.4	0.6	0.3	0.2	-	-	-	-	1401	1401	0
Isopropyl tetracosyl ether	-	-	-	-	0.7	-	-	-	-	-	-	2998	3000	2
**Ketones**	**Area (%)**													
Acetofenone	-	-	-	-	0.5	-	-	-	3.0	-	0.8	1061	1061	0
2 (3H) -furanone, 5-dodecildi-hidro-	1.2	3.6	5.1	-	2.0	5.4	2.7	3.6	5.4	6.2	4.6	2100	2104.3	4.3
**Terpenes**	**Area (%)**													
Lupenone	-	-	-	-	-	-	-	-	11.9	10.8	12.2	3384	3384.2	0,2
Lupeol	-	-	-	-	20.3	6.6	8.8	0.1	-	-	-	3499	3500	1
Lupeol acetate	16.4	17.5	10.6	6.1	3.0	3.4	3.7	0.2	-	-	-	3533	3525	−8
